# Menstrual Equity Initiatives at USA Universities: A Multiple Case Study of Common Obstacles and Enabling Factors

**DOI:** 10.3389/frph.2021.787277

**Published:** 2021-12-08

**Authors:** Caitlin Gruer, Taylor Goss, Margaret L. Schmitt, Marni Sommer

**Affiliations:** ^1^Department of Sociomedical Sciences, Mailman School of Public Health, Columbia University, New York, NY, United States; ^2^Department of Health Policy and Management, Mailman School of Public Health, Columbia University, New York, NY, United States

**Keywords:** menstruation, menstrual equity, period poverty, menstrual products, university, tertiary education

## Abstract

**Background:** In recent years there has been growing momentum in the USA around addressing issues of “menstrual equity” and “period poverty,” including a proliferation of university-level initiatives seeking to provide access to free menstrual products. This multiple case study examined four such efforts at a diversity of tertiary institutions to identify the factors that facilitated or impeded success.

**Methods:** We conducted a qualitative multiple case study, including a desk review and key informant interviews with student and administrative actors from universities with free menstrual product initiatives. We sought to identify key learning regarding common challenges and obstacles, enabling factors which supported success and sustainability, and practical learning for future initiatives. From the desk review, four schools (*n* = 4) were purposively selected to represent a range of geographic regions, student population size, and university type. Purposive sampling was used to identify students and administrators engaged in the menstrual equity initiatives on each campus (*n* = 20; 4–6 per school). Data from the desk review and interviews were analyzed using thematic analysis.

**Results:** Key themes included (1) the critical role of champions, (2) the importance of social and financial support, (3) challenges diffusing menstrual equity from pilot to scale, and (4) recommendations for future initiatives. University initiatives varied greatly in terms of their scope, funding, and implementation strategy.

**Conclusion:** This multiple case study provides valuable insights regarding the facilitating factors and obstacles faced by initiatives providing free menstrual products at universities. To date, these initiatives have proven successful across the four case studies; however, in most cases, the scope of the initiatives was constrained by limited resources and sustainability concerns. Future campus menstrual equity strategies would benefit from cross-institutional learning and dialogue highlighting design and implementation successes and challenges.

## Introduction

In recent years there have been growing reports in the United States of America (USA) of student-led initiatives to address period poverty and menstrual equity on university campuses, most commonly through the provision of free menstrual products (1, 2). Such initiatives emerged from the larger movement to address menstrual inequities across the USA and globally. These have included efforts to remove taxes on period products in the USA and other high-income countries in particular, and to tackle the menstruation-related challenges that girls face in school in low- and middle-income countries (3–5). The latter includes a lack of access to menstrual products, private and supportive bathroom facilities, menstrual health and hygiene (MHH) education, and inadequate support for menstrual pain and anxiety, all of which in turn negatively impact the health, well-being and educational experiences of menstruating students (3–8).

Although limited research exists on MHH in the USA, studies have identified vulnerable populations including girls in high-school (9), young women in college (10), low-income women (11), and people experiencing homelessness (12–14), all of whom regularly struggle to access menstrual products. In addition, there is a small but growing evidence base about the other challenges faced by menstruating students in the US that are very similar to those seen in low- and middle-income countries, such as inadequate MHH education, problematic school bathroom usage policies, and menstrual pain (10, 15–17). A parallel growing legislative movement in numerous cities and states seeks to provide access to menstrual products for vulnerable populations. For example, numerous states such as California, Illinois, Maryland, Oregon, Washington, and New York have passed legislation to provide girls in school with access to free menstrual products (18). Similarly, legislation mandating that homeless shelters provide menstrual products to their clients and that prisons provide free menstrual products to female inmates exists in a small but growing number of states (19). To date, however, there is minimal evaluation of the implementation and impact of these policy initiatives that would serve to inform future resource investment.

Less is known about the menstrual needs and challenges faced by students who menstruate at the university or college level. In the USA, “college” and “university” are often used interchangeably although colleges are typically smaller institutions that focus on undergraduate education, while universities tend to be larger institutions with both undergraduate and graduate education programs (20). This paper will use both terms depending on the language used by the specific institution to describe itself. One recent study conducted with 471 undergraduate university women across the USA found that 14.2% had been unable to afford the menstrual products they needed at some point in the past-year, and an additional 10% were unable to afford menstrual products every month (10). Inability to afford menstrual products differed significantly by race, immigration status, and familial college history. Latina and Black women reported period poverty within the last year more often than White women and women of another race (10). Similarly, those respondents born outside of the USA were more likely to report period poverty in the last year and the last month than those born in the USA, and first generation college students were more likely to have experienced period poverty than non-first generation students (10). Across the study sample, the inability to consistently afford menstrual products was associated with negative mental health outcomes, including depression (10). Although more evidence is needed, this study serves to highlight how access to menstrual products may be an important issue on numerous university campuses, a conclusion further shored up by the attention it has gained from student activists.

There is a long history of university-level student activism and advocacy in the USA, both on and off campus. This has illustratively included wartime opposition in the 1930s and 1960s, racial justice and desegregation in the 1940s and 1950s, the rights of women's and the lesbian, gay, bisexual, transgender, and queer (LGBTQ) communities in the 1960s, and for individuals experiencing homeless and hunger in the 1970s (21). Modern examples of student advocacy are often continuations of earlier fights for equality, including the growing number of menstrual equity advocacy initiatives occurring on university campuses today (21). These student-led menstrual equity movements combine prior advocacy issues related to gender, socioeconomic status, homelessness, and LGBTQ equity with a component of health and bodily autonomy that has historically not been directly addressed in campus activism (21, 22). Menstrual equity is an issue of gender equity and of social justice, assuring those with lesser means can access products and manage their periods with dignity and comfort.

This paper describes a multiple case study that was conducted using qualitative methods to examine four diverse student-led menstrual equity initiatives from across the USA. Given the large range of higher educational institutions in the USA (e.g., public, private, large or small student populations), it was important to explore these initiatives in varying contexts. The aim was to understand how these initiatives organized, mobilized financial and social support, and developed plans for sustainability.

## Materials and Methods

We conducted a qualitative multiple case study, including a desk review to explore existing university-based menstrual equity movements in the USA and key informant interviews (*n* = 20) with actors engaged in initiatives focused on the provision of free menstrual products on four diverse university campuses. The application of a multiple case study approach enabled us to compare the differences and similarities across initiatives. The desk review was used to identify the four case study schools, and through the subsequent interviews, we sought to identify key learning regarding common challenges and obstacles faced by the architects of these initiatives, enabling factors which supported success and sustainability, and practical learning for future initiatives.

All study procedures were approved by the Columbia University Medical Center Institutional Review Board.

### Desk Review

A desk review of the existing peer review and gray literature was conducted to gain an overall understanding of the menstrual health movement landscape at USA universities and enable the compilation of an initial list of universities with past or ongoing menstrual equity movements. This included a review of gray literature (news media, social media, internal reports) that described menstrual equity initiatives on university and college campuses. Google internet searches were conducted using a variety of search terms including “menstruation,” “menstrual,” “period,” “college,” and “university.” News media results related to the provision of menstrual products on university or college campuses were cataloged, yielding a list of 36 universities and colleges reporting a successful or proposed menstrual product initiative. For the purposes of this study, initiatives were considered successful if they had launched and were actively providing free menstrual products.

#### Selection of Four Case Study Schools

The 36 schools identified through the desk review were categorized by (1) type of institution (Public or Private), (2) USA geographical location, including Northeast, Southeast, Midwest, Southwest, and West Coast, and (3) undergraduate student population size. These categories were chosen to ensure that a diversity of types of schools and student body populations were represented. Of the 36 schools identified, there were 19 private schools and 17 public schools. The schools ranged in geographic location, with 16 located in the Northeast, 9 in the Southeast, 6 in the Midwest, 1 in the Southwest, and 2 on the West Coast. The schools varied in size from an undergraduate population of 2,000 to 55,000 students.

As shown in Table 1, four schools were then purposively selected to represent a range of geographical regions, undergraduate population size (7,000–32,000), and two private and two public institutions.

**Table 1:** Basic characteristics of selected case study schools.

	**A**	**B**	**C**	**D**
**School Type**	**Private**	**Private**	**Public**	**Public**
Undergrad Population	7,000	18,000	17,000	32,000
Geography	Midwest	Northeast	Southeast	West

#### Key Informant Interviews

Across the four campuses, key informant interviews were subsequently conducted with two groups of actors involved in the development and implementation of the menstrual equity initiatives. This included *Group 1*, comprised of students engaged in an existing campaign (*n* = 10); and *Group 2* which included campus administration, faculty, and staff (*n* = 10). Table 2 shows the breakdown of key informants by group and university.

**Table 2:** Study participants by university and group.

**University Code**	**Group 1**	**Group 2**
A	3	2
B	2	4
C	2	2
D	3	2

Two semi-structured interview guides were developed to capture the range of participant's expertise, knowledge, and engagement in the menstrual equity initiatives. The Group 1 guide explored the experience of campaigning for campus menstrual equity, including the student's motivation for engaging in the topic and the process, successes, and challenges of spearheading such an initiative. The Group 2 guide focused on the administrative perspectives of menstrual equity campaigns, why they supported the campaigns, the challenges they faced, and what hesitations they had for implementing a program for increased menstrual product access.

#### Sampling and Recruitment

Key informants were selected across the four schools utilizing purposive sampling (*n* = 20). Participants were identified based on their past or present involvement in initiating, implementing, or supporting an existing menstrual equity initiative. Initial participants were identified through the desk review. Recommendations were solicited from the initial participants to generate a list of additional actors for inclusion. The researchers recruited potential participants via email or telephone. Individuals who responded were provided with background on the study and invited to participate in an interview.

#### Data Collection

Three researchers conducted semi-structured qualitative interviews with the 20 key informants in-person, over the phone and via videoconferencing (Zoom) to accommodate schedules, geographic distances, the social distancing constraints of the COVID-19 pandemic, and participant preference. Data collection took place between November 2019 and April 2020. Interviews ranged from 20 to 60 min in length and were recorded after receiving informed consent from the participant. The recordings were transcribed for analysis.

#### Data Analysis

Two researchers reviewed the documents from the desk review and the written transcripts from the qualitative interviews. Malterud's “systematic text condensation” method for qualitative analysis was used to identify key themes of interest (23). This included the following steps: (1) identification of preliminary themes; (2) creative development of qualitative codes; (3) condensation of coded text; (4) synthesis and reconceptualization (23). A simple codebook of the key themes was developed, and Dedoose qualitative analysis software was used to code the interviews. The key themes identified in the data were shared with the larger research team for discussion, refinement and validation (23). The key analytical themes identified during analysis are presented below, along with illustrative interview excerpts.

## Results

Each of the universities in the study had a menstrual equity initiative providing free menstrual products operating on their campus. Table 3 provides an overview of the university demographics and initiative characteristics, including the process for establishing the initiative, the budget source, the labor and maintenance source, and the locations targeted for distributing the free products. When defining the locations of menstrual product distribution, participants used the terms “bathroom” and “restroom” interchangeably; however, for the purposes of this paper, the term “bathroom” is used throughout to refer to sanitation spaces housing both toilets and handwashing facilities.

**Table 3:** Characteristics of the menstrual equity initiatives across the four case study universities.

**Characteristic**	**University code**			
	**A**	**B**	**C**	**D**
**University characteristics**				
School Type	Private, religious	Private	Public	Public
Undergrad Population	7,000 (small/S)	18,000 (medium/M)	17,000 (medium/M)	32,000 (large/L)
Geography	Midwest (MW)	Northeast (NE)	Southeast (SE)	West (W)
**Menstrual equity initiative characteristics**				
Process	Student proposal for an annual autonomous wellness grant approved by the student government	Student developed a proposal for the administration with support from a school-run entrepreneurial think tank	Students gained administrative support and administrators began a small-scale implementation	Student government developed a referendum to increase the student wellness fee to cover the cost of menstrual products and other initiatives
Budget Source	Annual wellness grant	Administration	Administration	Student wellness fees
Labor Source	Student-run volunteer group	Custodial/facilities staff	Custodial/facilities staff	Joint (students + custodial)
Recruitment Methods	Social media; informational meetings; word of mouth	High student involvement from the beginning including multiple student groups and student governments	Partnered with campus sorority leadership	Social media-based campaigning for referendum votes
Primary Champions	2 student leaders with administrative connections; 1 staff member	Multiple student government leaders; 1 administrator; campus think tank	2 student leaders; 1 facilities administrator	Multiple student government leaders; on-campus student health center admin
Locations	10 classroom buildings plus recreation center, ~20 bathrooms	5 major classroom buildings, 160 bathrooms (undergraduate campus only)	3 buildings (2 major common areas and 1 large classroom building), 18 bathrooms	Health Center, LGBTQ Center, housing offices of some dorm buildings, and some libraries
Type of Location	Female-assigned and single-use bathrooms	Female-assigned, male-assigned, and single-use bathrooms	Female-assigned bathrooms	Outside of bathrooms and in common areas
Initiative Status	Student implementation and student upkeep; some struggles due to dependency on student volunteer availability	Staged rollout by university; permanent funding source unclear	University facilities-run pilot; searching for permanent funding	Joint rollout in select locations
Unique Features	Entirely student-run; utilized commercial menstrual product company for advice and supplies	Proposal supported by entrepreneurial think tank; city passed a menstrual equity bill during student movement	Strong administrative support and leadership	Initiative is now institutionalized; disjointed facilities permissions per building

The specific mechanisms and implementation processes of the menstrual equity initiatives differed across the four schools, however patterns emerged around four thematic areas: (1) the critical role of champions, (2) the importance of social and financial support, (3) challenges diffusing menstrual equity from pilot to scale, and (4) recommendations for future initiatives.

### Critical Role of Champions

Across all four schools, champions played a critical role in the success of these initiatives from initiation through implementation. In all cases, the menstrual equity initiative was initiated and championed by undergraduate students, typically with one to two students identifiable by fellow students and administrators as the initiative leaders. These student champions were primarily motivated by what they perceived to be an important issue of inequality. While some champions were motivated by a personal interest in reproductive health and women's health issues, most were extrinsically motivated by stories from their fellow classmates about challenges accessing menstrual products on campus or news articles about period poverty. As a student champion from School A (S, Private, MW) described:

…*we had someone reach out to us via email, basically explaining her story about how as a commuter student 1 day she had gotten her period and was basically unable—she didn't have any menstrual products on her and there were none available at the school. And so, she essentially had to go home for the day and it interrupted her education. –KII 16*

The presence of one to two clear student leaders associated with the initiative seemed to facilitate success, providing unity to the student's messaging and a singular point of contact for conversations between the administration and student body. Conversely, a lack of unity posed a significant threat to success. Before the existing sustained menstrual equity initiative at School B (M, Private, NW), there were a number of unsuccessful initiatives that emerged from various student groups to promote free menstrual products. While there was reported to have been significant interest among the student groups, there was a lack of unity across these differing but simultaneous efforts, leading to fragmentation and stagnation. When the multiple student initiatives were instead united under one clear student leader or champion, the initiative was found to be more successful in communicating with the administration and furthering the overall goal of advocating for free menstrual products on campus.

At all four schools, the student champions were members of the student government. These positions enabled them to build student support, and to engage with the university administration to move the initiative forward. The student participants reported that their role in student government facilitated their success in championing the menstrual equity initiative as it provided unique access to members of the administration and a strong understanding of the university bureaucratic systems. As one of the student champions from School C (M, Public, SE) described:

*I was pretty well versed in university budget issues, you know, school budgets, division budgets, and that kind of stuff, you know what the bureaucracies were, when deadlines had to be made, something that most students and particularly student leaders were never quite interested or capable of delving into. –KII 13*

While the student champions played an integral role in initiating and developing support for the idea of a menstrual equity initiative, their role in obtaining and managing funding and implementation differed across schools. For example, at School A (S, Private, MW) the two student champions not only conceived the idea for the initiative and ran the pilot, but they also acquired funding via a student government wellness grant to implement the initiative, and, with a small team of other students, were responsible for stocking the menstrual products in the dispensers across campus. Similarly, at School D (L, Public, W), the student champions were able to pass a referendum through student elections to levy a small increase to the student wellness fee to cover the cost of the free menstrual products, with little need for input from the administration. However, at School C (M, Public, SE) and School B (M, Private, NE), the student champions were heavily involved in the conception and initiation of the initiative, but the administration was primarily responsible for funding and implementation.

The support of a champion within the staff or administration was also a crucial facilitating factor in generating buy-in and support beyond the student body in three of the four schools. The exception to this was School D (L, Public, W) where the need for an administration champion was less crucial due to the ability of the student government to cover the cost of the initiative by levying stable and recurring funds via student fees. The role of these administration champions varied, and included securing funding, helping students to navigate the university bureaucracy, providing guidance on proposal development, and gathering support and approval from other members of the university bureaucracy. As a staff member from School B (M, Private, NE) described:

*I think they needed a champion on campus, so I think [high level administrator] helped play that. He heard the students, he heard their concerns, he recognized their perspective that this was a human rights issue, and he became a conduit to connect them with facilities. –KII 11*

Across the cases, this administration champion was seemingly more effective when they were more senior, had budgetary control, and were better-connected to senior leadership whose approval and buy-in was required for implementation (e.g., operations or facilities staff).

### Importance of Social and Financial Support

Critical to the success of all four menstrual equity initiatives was obtaining social and financial support, with student champions describing the importance of generating support from both the student body and the administration. As one student respondent explained:

*I think the biggest roadblock was getting the stakeholders on board because without their support, I could fight for like 3 years and they were never going to—like they could have just never, never taken it on and then it wouldn't have happened. –KII 04*

To generate initial support, the student champions utilized numerous methods, including surveys of the student body to ascertain level of need, petitions to document the extent of student body support, and campus-level social media campaigns to generate awareness and mobilization for the initiative. The student champions noted a difference in how certain audiences responded to various strategies of framing the issue of menstrual equity. While undergraduate students seemed more responsive to language that framed the issue as one of equity, social justice, or morality, university administrations were generally more responsive to proposals framing the initiative as addressing period-related emergencies (e.g., an unexpected menstrual period or running out of supplies). As one student described:

*I think when I was trying to market the program to students and things like that, we're definitely kind of making it more like “This is a necessity. We recognize this is a necessity. Please take it because this is something that is just systemically wrong and needs to be addressed.” But definitely in terms of like the administration, like we kind of talked about this moral argument and we kind of said that like, “yes, this is a necessity and people need it.” …but kind of framing it almost as in an emergency situation for them to feel like, “oh, people aren't going to just let's take it.” And like the “if you need them” kind of thing was kind of our approach. So, I definitely took different framing depending on the population you were talking to. –KII 06*

In addition, at each school, the students conducted a student-run pilot, typically consisting of providing free menstrual products from a basket in a small number of centralized locations on campus. These pilot programs were typically implemented by the students themselves, including the restocking of the menstrual products, and were funded via discretionary funds from student government or other student body organization budgets. These small pilots were used in conversations with administration and the broader student body to demonstrate both the need and usefulness of the products by the campus population, as well as proof of concept. In some cases, these pilots also proved to mitigate fears of misuse, as in most cases there was no evidence of tampering with the supplies, or of students taking an excess number of products.

Despite these measures, there was some resistance to the menstrual equity initiatives across all four campuses. Most frequently the opposition came from members of the administration. As one administrator explained:

*There were like a couple people that I encountered that were kind of like “this isn't important, or this isn't something that the whole student body can benefit from, so you shouldn't be spending your time on it.” —KII 10*

Similarly, some administrators expressed concern about the viability of a free product distribution system, including the potential for vandalism of the dispensers or misuse of the free products. As a student from School D (L, Public, W) described:

…*there was a lot of concern that students would steal these products…when they were made available for free. They were like, 'oh, what if, you know, somebody takes the whole box or someone just like decides to walk off with all the products' and we were like “well doesn't that mean that people need them?” —KII 03*

Although there were very limited reports of vandalism or product misuse during the implementation of the initiatives, these detractors had to be either won over or overruled before the initiatives could progress to the point of implementation. Addressing this opposition from the administration required understanding and addressing their concerns. This often required stepping away from the idealism or moral framing of the issue of menstrual equity. As one administrator described:

*[The students] said, “Well, this is just right. Everybody should be into it and want to do it.” And I had to say, “Well, not necessarily. Not everybody sees it your way. And look, I'm behind you but part of being behind you means we've got to educate the community.” –KII 19*

In addition, one of the most common concerns was the potential cost of the initiative. Across the universities, students and administrators alike struggled to estimate the real implementation costs. While administrators tended to overestimate how much the initiatives would cost, student's initial budgets often failed to account for less visible expenses such as the cost of labor to maintain the stock of the menstrual products and maintenance of the dispensers in cases of damage or malfunction. This underscores the necessity of a detailed, evidence-based budget proposal that accounts for all associated resource requirements, and the importance of good collaboration between the students and the administration.

### Challenges Diffusing Menstrual Equity From Pilot to Scale

Although the pilot projects were all successful, each school faced significant challenges in relation to diffusing the menstrual equity initiatives from the small number of locations included in the pilot to a more widespread distribution due to the increased funding, labor, and maintenance requirements when operating at scale.

During the effort to move beyond the initial pilots to operate in more locations across campus, each initiative had to determine the source of labor for distributing and maintaining a consistent stock of menstrual products across the target locations, with the two primary options being the custodial staff or the student groups. Each option presented benefits and drawbacks, as relying on the custodial staff required the buy-in and support of multiple levels of the administration, while student-managed systems placed a large burden on the students responsible. Three of the schools (B, C and D) opted to work with the administration to utilize staff-managed distribution systems. As a student from School D (L, Public, W) described, this was not the fastest or easiest option, but was more sustainable over time:

*I think one of the biggest issues that we faced after the funding was secured was just getting the administrative buy-in…But I think that was one of the things that really made it successful, because if we don't really have the buy-in then, it becomes a little bit tougher to get this kind of rolled out because…there are a decent amount of us [students] but there's also not enough to continuously replenish all the sources like, yes, we can buy them and then we can go and distribute it at the center, but we can't really be there every single day to replenish it. So that kind of takes a lot of administrative buy-in. –KII 06*

In line with this, School A (S, Private, MW), which utilized a student-run system, encountered implementation challenges due to competing student priorities and turnover of the student leaders with no clear successor. While the students were typically able to maintain the menstrual products stock, it constrained the number of locations in which the initiative could be feasibly implemented and placed significant stress on the student implementers.

Due to resource limitations, both financial and human, each of the initiatives were forced to make difficult decisions and compromises about where the free products would be located. While students across all four universities began with the idea that all bathrooms or all locations across the school should have free menstrual products, this was challenging in practice due to budget and labor constraints. Instead, all four initiatives focused on centrally located or high-trafficked buildings with the intention of making the menstrual products as accessible as possible.

Also central to this decision was a discussion about what types of bathrooms (i.e., female-assigned, male-assigned, or gender-neutral) should be targeted for the free product distributions. This included deciding whether it should be those frequented by the highest concentration of people who menstruate (i.e., female-assigned bathrooms), or an equal number of female- and male-assigned or single-use bathrooms to meet the needs of all people who menstruate. Student champions from all four universities initially proposed universal implementation, targeting gender-neutral, single-use bathrooms, and/or male-assigned bathrooms in addition to female-assigned bathrooms. As one of the student champions described:

*I personally came into the room saying “This should be in every single restroom, unisex, men's restrooms, and women's restroom. They should be in every single restroom.”—KII 13*

However, in some cases, these proposals faced resistance from members of administration who questioned whether the level of need in male-assigned locations justified the required maintenance and upkeep. As an administrator from School B (M, Private, NE) described:

…*we provide products in all of the single-occupant because they're gender-neutral…The other half of them that are multi-occupant restrooms, I have about eight hundred of them and if I assume that half of them are currently labeled men and the other half are currently labeled women, those four hundred [that are labeled men] are the ones that I'm curious about whether or not we need to supply all of them. –KII 10*

Similarly, some administrators expressed concern that misuse of products would be more common in male-assigned bathrooms due to curiosity and lack of understanding, although there are conflicting reports of whether this concern was valid based on actual implementation experiences.

Ultimately, the initiatives were forced to make compromises in terms of what locations would be included in the distribution, due to budgetary constraints and in some cases administrative opposition. As one student described the trade-offs involved in this discussion:

…*[inclusivity] is like a two-edged sword and it's awful, but not being able to put them in the male-assigned restrooms because we were able to spread them out to more buildings. So, we were able to include them in the school of engineering, which is something we wouldn't have been able to do. We were able to put four on the med campus instead of just two. So that definitely helped us out. But it wasn't kind of in the way that we wanted, but it is definitely the overall distribution we needed. But that was a hard thing. We also chose to put them only in academic buildings and the student union. –KII 01*

At the time of data collection, three out of the four universities had implemented some form of inclusive distribution: University D (L, public, W) implemented public dispensers outside of bathrooms that could be accessed regardless of the gender-assignment of the bathroom used; University A (S, private, MW) installed formal dispensers inside single-use bathrooms; and University B (M, private, NE) was perhaps the most inclusive by providing menstrual products in all bathrooms (female-assigned, male-assigned, and gender neutral) in the selected buildings.

### Recommendations for Future Initiatives

The student and administration respondents shared a number of ideas for other schools or student champions considering initiating similar projects to provide access to free menstrual products. The most common recommendations made by student participants were inspirational sentiments surrounding the amount of hard work a project like this might entail but encouraging future advocates to remain dedicated and resilient.

*So, yeah, the advice is you should really just do it. I think it will really, really be something that, you know, is necessary…So I think there is a lot of momentum specifically right now for these projects to kind of take flight. And I do think students should take advantage of it and really use all these initiatives and these bills of laws to really kind of swing that and just, you know, start the project. —KII 07*

Notably, almost every student respondent mentioned the immense time commitment required to champion one of these initiatives, a commitment that should not be overlooked by student champions seeking to undertake similar initiatives. To increase the feasibility and chances of success, one student champion recommended utilizing the student government structures, even if the champion is not a member of student government.

…*really talk to student leaders and hold them accountable. I think that's something that's important. Being a student leader, if that one student hadn't reached out to [us as members of student government], we would have never started this, you know? So, I think, if you're on the other end, be the person that's like, “Hey, why aren't you doing something about this or let's get together and do something?” –KII 16*

This commitment did appear to be less significant in Schools B and C in which the administration was more involved in the pilot and implementation processes, indicating that the required student commitment could be mitigated by earlier administration involvement.

In addition, many of the student respondents recommended considering how inclusivity could be incorporated into the initiatives, including ensuring access to menstrual products for transgender and non-binary student populations and avoiding the use of gendered language, such as “feminine hygiene products.”

A second set of recommendations, primarily offered by the administration respondents, focused on practical considerations for gaining support from key stakeholders with political, budgetary, and implementation power. These recommendations centered on understanding the university context and constraints, so that any initiative could be designed to address or avoid them. While these recommendations were mostly directed toward prospective student champions, they could also be relevant to more junior administrators interested in implementing free menstrual products on campus–although the desk review did not uncover any examples of an administrator initiating such advocacy without pressure from students. As one administrator recommended:

*Approach this as a question of why do we currently—what were the constraints that led to us not providing these products? And have they changed? And if so, what are others doing that they've figured out? —KII 10*

This includes the use of data and evidence from both the student body and other initiatives, and a well-conceived and researched proposal. As an administrator from School B (M, Private, NE) suggested, this is not only critical for generating consensus but also for overcoming any opposition:

…*the best advice I could give is that the only way to fight misperception is with data. And the more fact-based, one can be in the discussion—about costs, about availability, about theft, about all these sorts of petty excuses that people use not to do something—the better off you are, the more strength of the argument is. —KII 09*

Additional recommendations from administrative participants included finding an administrative champion, being realistic about funding expectations and increasing student body awareness about the initiative to gain support and aid students in understanding the rationale for menstrual product dispensers in male-assigned bathrooms.

Finally, both the administration and student respondents emphasized that it was critical to think about the sustainability of the initiative from the onset to ensure that the initiative was designed in a manner that allows for and promotes success in the long-term. As one student respondent described:

…*there needs to be longevity to it, like you need to make it a sustainable program. And so, you need to think about more than just right now and how will it work. Even if that's like the starting point, there has to be some way for it to continue beyond you and whatever group you're working with. –KII 03*

Sustainability was primarily described in terms of obtaining a permanent source of funding to maintain access to free menstrual products, administrative oversight of the stocking and maintenance of the dispensers, and potentially expanding free menstrual product access to more buildings or bathrooms.

## Discussion

Menstrual equity initiatives on university campuses are appearing with increasing regularity (24), and likely will continue to do so as attention to menstrual equity and period poverty continues to gain momentum in the USA and around the world. These initiatives may be particularly pressing as more reports emerge documenting the challenges that many college students, particularly students of color, face in accessing and affording basic necessities, including menstrual products (10, 25, 26). The case studies shared here provide important insights into common challenges and enabling factors encountered by students and administrators seeking to enact this type of programming, providing critical learning for other localities and stakeholders seeking to create and implement similar initiatives. Importantly, these initiatives were all launched by students, however as recognition of menstrual equity gains attention, universities may themselves initiate policies equipping all on-campus bathrooms with menstrual products. This study was not able to extract any patterns based on university type (private/public), geography, or size. The budget source, size and scope of the four initiatives studied varied across types with no within-group comparisons for type, geography or size.

Overall, the four case studies illustrate the critical importance of having champions both within the student body and the administration. These key players were necessary for generating support, navigating the university bureaucracy, and providing a singular point of contact for the administration or other students to direct questions and concerns. In some cases, the centrality of the student champion to the initiative implementation proved problematic when they graduated, studied abroad, or became preoccupied with other priorities. The effectiveness of a champion is evident within the broader menstrual equity movement as well. In Scotland, the grassroots efforts to address period poverty nationwide were spearheaded in part by Victoria Heany who started the #FreePeriodScotland campaign and garnered sufficient interest for the issue to capture the attention of the Scottish government, leading to the eventual passage of legislation mandating universal access to menstrual products across the country, the first legislation of its kind globally (27–29). Also critical was a staunch champion within the Scottish parliament, Monica Lennon, who took up the issue, introduced the bill to parliament, and advocated for the legislation internally until its passage (27).

The findings also highlight the importance of generating both social and financial support and the multiple methods that can be used to do this, such as small-scale pilot projects to show proof of concept, surveys to demonstrate need, and advocacy and social media campaigns to generate interest. These approaches are important to consider not only within university settings, but also at the state or national level as they have also been found to be effective in the push for broader menstrual equity legislation. For example, during the menstrual equity campaign in Scotland, activists developed a multi-pronged campaign utilizing surveys, testimonies from Scottish women and girls, social media, and a pilot initiative to not only provide evidence of need but also to generate and showcase broad public support for the effort (30, 31). Also critical was the ability to tailor the message framing as needed, with the student champions modifying their messaging as needed to be most compelling for their target audience; equity was the main concern for student peers while “emergency-use” was a more convincing argument for many administrators.

Similarly, multiple localities have utilized small-scale pilots to test the feasibility and impact of initiatives to provide free menstrual products (32, 33). For example, before mandating that schools across the city provide access to free menstrual products to schoolgirls, New York City conducted a small-scale pilot first at one school, and then in 25 middle- and high- schools in primarily low-income neighborhoods in two areas of the city (34, 35). In addition to providing evidence of the positive impact, these small-scale pilots can also be useful to mitigate fears of misuse or potential hoarding of the menstrual products (36). Fears of vandalism or misuse are common across contexts; however, there have been few examples of these challenges to date, with those that have occurred primarily limited to the free products being thrown out in male-assigned restrooms (37, 38). Future advocates should gather supporting evidence for their cause, including but not limited to figures from similar universities with successful initiatives and internal figures from within the university about student body need and interest. Although only one of the case study universities utilized evidence from another university, external experience, including practical lessons learned, may serve as a critical source of information and guidance.

These case studies also draw attention to some of the common challenges to the successful initiation of a menstrual equity initiative. Two frequently identified hurdles included addressing opposition from members of the administration and securing a sustainable and sufficient source of funding. In some cases, well-developed proposals can be used to overcome these challenges, particularly when they include details regarding the size and scope, timeline, labor and product sources; however, initiative leaders must also be flexible and open to the suggestions and compromises offered by other stakeholders. This is evident within the broader menstrual equity movement as well. For example, while the proposed Scottish legislation was very detailed in terms of the scope, timeline and proposed budget, the policy was still amended to simplify, clarify and address the concerns of some Scottish Ministers (39). Similarly, in Brazil, youth activists developed a period poverty policy proposal that they then shared with local officials. In the state of Rio de Janeiro, this proposal attracted the attention of a state representative who became a staunch advocate, leading to the passage of a bill to reduce the tax on menstrual products and make them more accessible (40).

The resource challenges within the four school initiatives raised important conversations around diversity and inclusion, such as whether male-assigned bathrooms would be included in the distribution scheme to meet the needs of transgender or gender non-binary (TGNB) members of the student body. A recent study found that menstruating TGNB individuals who were assigned female at birth (AFAB) described female toilets as confrontational spaces where they receive incriminating stares and body policing (41). As such, to meet the needs of these individuals, it may be particularly important to provide access to menstrual products in locations beyond female-assigned bathrooms. These conversations are part of a larger effort by universities, brought on by growing social pressure, to create more inclusive environments and meet the needs of TGNB students. This has included for example, the hundreds of colleges and universities that have added gender identity and/or gender expression to their policies on non-discrimination (42), and the hundreds who have created or are in the process of creating gender-inclusive restrooms on their campus (43–45). These conversations were also prevalent within this study's sample, with the administration participants from multiple schools highlighting the ongoing initiatives on their campus to increase the availability of single-use bathrooms or to create “all-gendered” or “gender neutral” bathrooms spaces. It is important to note that despite these ongoing conversations and efforts toward creating inclusive environments for TGNB students, overt and covert discrimination is still present on university campuses and further work is needed in this area of MHH research (42).

While the number of these university and college menstrual equity initiatives seems to be on the rise, there is also a simultaneous mobilization for legislation mandating that public universities provide free menstrual products to their students. Already this legislation was passed in Scotland (29) and the United Kingdom in 2020 (46) and in several states in the USA (e.g., Washington State, Illinois, California) in 2021 (47–49). Although it remains unclear how these mandates will be operationalized or monitored, they represent an important step toward the standardized integration of menstrual product provision into normal campus operations. This legislation may render the campus-level initiatives described in this study unnecessary as it seeks to institutionalize this practice at the state or national level. However, these initiatives may provide useful guidance to policymakers and administrators in the states with these mandates on the cost, implementation models, distribution schemes, and challenges of providing free menstrual products on campus.

### Limitations

There are three limitations to note. First, although the study included four diverse universities, there are numerous other university student-led menstrual equity initiatives which might have varying factors impacting their implementation. Second, this study only explored successful initiatives, and so may miss important challenges or obstacles that caused other such initiatives to fail. Third, given the relatively recent and short period of menstrual equity initiatives on the four campuses, it was not possible to assess their longer-term sustainability.

## Conclusion

The examined university menstrual equity initiatives varied greatly in their scope, funding, maintenance, and labor sources, yet all four noted the presence of student champions, the importance of gaining administrative support, and the necessity of addressing inclusivity. Three recommendations emerge: (1) sustainable menstrual equity initiatives are more likely to occur when university administration commits resources and capacity to assuring menstrual products are widely available, (2) more research is needed to understand the most effective implementation approaches for menstrual equity initiatives on diverse campuses, and (3) additional learning is needed to understand how such initiatives may positively or negatively impact TGNB students, students of color, and those experiencing homelessness while in university. Finally, these menstrual equity initiatives play an important role in meeting the needs of students on college and university campuses; however, to fully address the issue of menstrual equity, structural solutions are required such as the institutionalized provision of menstrual products within all university bathrooms.

## Data Availability Statement

The raw data supporting the conclusions of this article will be made available by the authors, without undue reservation.

## Ethics Statement

This study involving human participants was reviewed and approved by the Columbia University Medical Center Institutional Review Board. Written informed consent for participation was not required for this study in accordance with the national legislation and the institutional requirements.

## Author Contributions

CG participated in study conception, conducted the data collection and analysis, and drafted the manuscript. TG participated in study conception, conducted the data collection and analysis, and contributed to drafting the manuscript. MS and MLS conceived the study and contributed to the drafting the manuscript. All authors read and approved the final manuscript.

## Funding

This project was funded with generous support by the Sid and Helaine Lerner MHM Faculty Support Fund.

## Conflict of Interest

The authors declare that the research was conducted in the absence of any commercial or financial relationships that could be construed as a potential conflict of interest.

## Publisher's Note

All claims expressed in this article are solely those of the authors and do not necessarily represent those of their affiliated organizations, or those of the publisher, the editors and the reviewers. Any product that may be evaluated in this article, or claim that may be made by its manufacturer, is not guaranteed or endorsed by the publisher.
